# SNP mutation‐related genes in breast cancer for monitoring and prognosis of patients: A study based on the TCGA database

**DOI:** 10.1002/cam4.2065

**Published:** 2019-03-18

**Authors:** Chundi Gao, Jing Zhuang, Chao Zhou, Huayao Li, Cun Liu, Lijuan Liu, Fubin Feng, Ruijuan Liu, Changgang Sun

**Affiliations:** ^1^ College of First Clinical Medicine Shandong University of Traditional Chinese Medicine Jinan PR China; ^2^ Department of Oncology Weifang Traditional Chinese Hospital Weifang PR China; ^3^ Department of Oncology Affilited Hospital of Weifang Medical University Weifang PR China; ^4^ College of Traditional Chinese Medicine Shandong University of Traditional Chinese Medicine Jinan PR China

**Keywords:** bioinformatics analysis, biomarkers, breast cancer, prognosis, single nucleotide polymorphisms

## Abstract

Advances in cancer biology have allowed early diagnosis and more comprehensive treatment of breast cancer (BC). However, it remains the most common cause of cancer death in women worldwide because of its strong invasiveness and metastasis. In‐depth study of the molecular pathogenesis of BC and of relevant prognostic markers would improve the quality of life and prognosis of patients. In this study, bioinformatics analysis of SNP‐related data from BC patients provided in the TCGA database revealed that six mutant genes (*NCOR1, GATA3, CDH1, ATM, AKT1,* and *PTEN*) were significantly associated with the corresponding expression levels of the proteins. The proteins were involved in multiple pathways related to the development of cancer, including the PI3K‐Akt signaling pathway, pertinent microRNAs, and the MAPK signaling pathway. In addition, overall survival and recurrence‐free survival analysis revealed the close associations of the expression of *GATA3*, *NCOR1*, *CDH1,* and *ATM* with survival of BC patients. Therefore, detecting these gene mutations and exploring their corresponding expression could be valuable in predicting the prognosis of patients. The results of the high‐throughput data mining provide important fundamental bioinformatics information and a relevant theoretical basis for further exploring the molecular pathogenesis of BC and assessing the prognosis of patients.

## 1 INTRODUCTION

Breast cancer (BC) is one of the most common cancers among women, and its morbidity and mortality have continued to increase worldwide in recent years, reflecting the strong invasiveness and metastasis characteristics of this cancer.^1^ BC is a complex disease that involves a sequence of genetic, epigenetic, and phenotypic changes. Polymorphisms of genes involved in multiple biological pathways have been identified as potential risks of BC.^2^ These genetic polymorphisms further lead to differences in disease susceptibility and severity among individuals.^3^ The development of accurate molecular diagnoses and biological indicators of prognosis are crucial for individualized and precise treatment of BC patients.

Bioinformatics analysis based on high‐throughput sequencing is an important method to explore the molecular mechanisms of tumor pathogenesis, identify biomarkers that permit early diagnosis, and discover therapeutic targets. Single nucleotide polymorphisms (SNPs) are DNA sequence polymorphisms caused by a single nucleotide variation. SNPs are the most common type of human heritable variation and are common in the human genome. Gene SNPs can cause changes in gene expression by affecting the binding, cleavage, methylation, and mRNA degradation of gene transcription factors, causing genetic differences among individuals.^4^ SNPs are considered potential markers of carcinogenesis, and thus are valuable for early diagnosis and personalized targeted therapy for cancer. Even more profoundly, the detection of SNPs linked to cancer may lead to the reversal of the malignant transformation of cells if these SNPs can be corrected.

As a relatively small allelic variable, an SNP is an important genetic marker to study the characteristics of different cancers or cancers. As genome‐wide association studies have progressed, there is an increasing evidence that BC susceptibility is associated with genetic SNPs. For example, SNPs of ERCC5 have been associated with the development of certain cancers, including BC.^5^ Nari et al showed that the ERCC5 rs2094258 polymorphism might damage the DNA repair mechanism by causing defects in nucleotide excision repair, which is closely related to the risk of BC.^6^ In addition, Sun et al found that polymorphisms caused by genetic variation of microRNA (miR)‐124 rs531564 affect the prognosis of cancer patients.^7^ As SNPs associated with cancer risk may affect prognosis, analysis of relevant SNPs may help to identify new biomarkers for the prognosis of cancer.

The Cancer Genome Atlas (TCGA) database can be applied to high‐throughput genomic analyses to better demonstrate the genetic basis of disease by using genome sequencing and bioinformatics analysis of gene mutations responsible for cancer. The findings could improve our ability to diagnose, treat, and prevent cancer. To further explore the biological significance of DNA sequence polymorphisms in the diagnosis and prognosis of BC, we downloaded data of BC‐related SNPs from the TCGA database and used bioinformatics analysis methods, including mutation data, protein‐protein interaction (PPI) network, and correlation analyses, to mine mutation genes related to BC diagnosis and prognosis. The goal was to provide a scientific theoretical basis for personalized precision medical treatment for BC.

## 2 MATERIALS AND METHODS

### 2.1 Data processing and analysis

TCGA Data Portal was terminated, and all TCGA data were transferred to the newly established Genomic Data Commons (https://gdc.cancer.gov/).^8^ As the raw data on SNP in TCGA are not open to the public, we downloaded the SNP‐related data of BC that has been processed along and the raw mRNA expression data. The mRNA data were compiled from 1208 samples, including 112 normal samples and 1096 cancer samples. The mutated gene was obtained from the downloaded BC sample SNP data. The downloaded mRNA raw data were integrated and standardized using the Edger software package, and differences were analyzed to obtain differentially expressed genes and their expression level. The mRNA data provided by TCGA are public and open‐ended, and therefore does not require the approval of a local ethics committee.

### 2.2 Functional enrichment and pathway analysis of mutant genes

To better understand the dysfunction caused by these mutant genes, we used the DAVID (https://david.ncifcrf.gov/)^9^ database to perform agonistic gene ontology (GO) and Kyoto Gene and Genome Encyclopedia (KEGG) enrichment analyses on genes with more than 15 mutant samples. As an open source platform, DAVID can be used to determine the association between target molecules. By selecting the GO term and the KEGG pathway and using *P* < 0.05 as the cutoff condition, screening of molecular functions (MFs), biological processes, cellular components (CCs), and KEGG pathways for mutated gene enrichment can be accomplished.

### 2.3 Construction of mutant gene PPI network and gene expression analysis

The construction of biological networks can be extended in the form of an actual system scale, and provides a visual representation of molecular interactions. We used the STRING online database^10^ to characterize the PPI network of the mutant genes and set the confidence score >0.4 as the cutoff criterion. We visualized the generated PPI network using Cytoscape software.^11^ To analyze the role of mutations in the development of BC, we explored the correlation between mutations and gene expression. In addition, the relationships between the site that were mutated in more than two samples and gene expression were further explored.

### 2.4 Mapping of Kaplan‐Meier survival curve of mutant genes and screening of prognostic biomarkers

The Kaplan‐Meier plot can evaluate the survival of breast, lung, stomach, and ovarian cancer patients using the gene expression data. Recurrence‐free survival (RFS) and overall survival (OS) data were downloaded from GEO (Affymetrix microarrays only), EGA, and TCGA. The primary purpose of the tool is a meta‐analysis‐based biomarker assessment.^12^ Using the Kaplan‐Meier plot, we evaluated the effects of mutant genes on the prognosis of BC patients, and finally screened for mutated genes that could be used as prognostic biomarkers for BC.

## 3 RESULTS

### 3.1 Data processing and analysis

**Figure 1:**
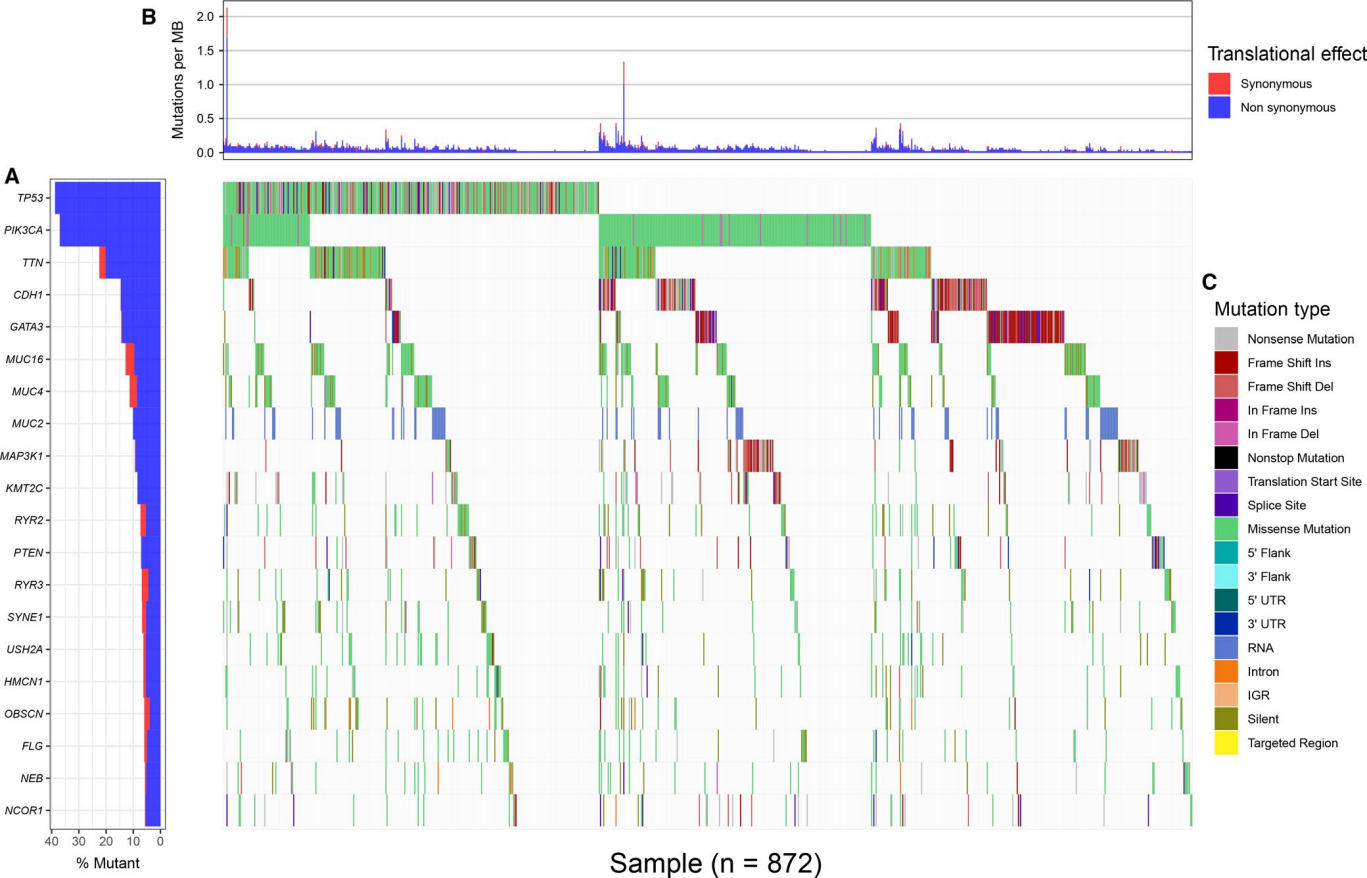
A waterfall map of 20 genes that mutated in more than 50 samples. (A)mutated gene, (B)tanslational effect, and (C)mutation type

**Figure 2:**
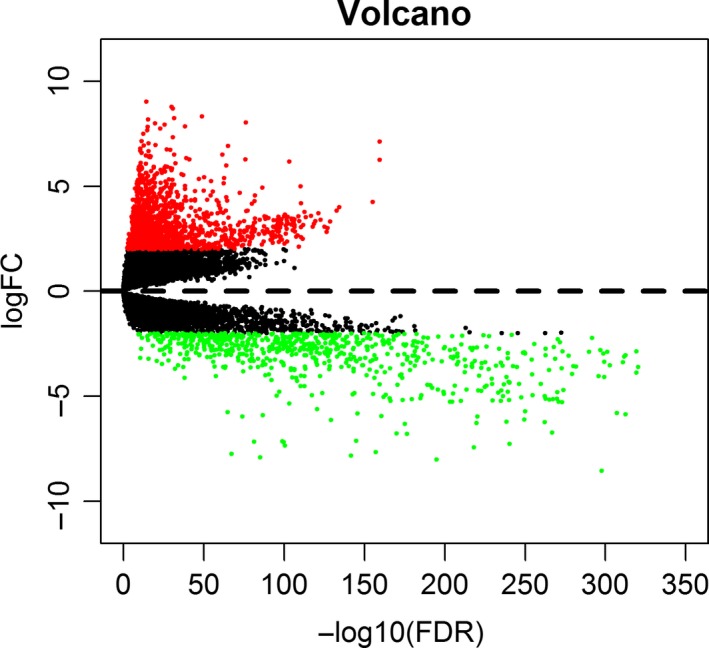
The volcano diagram about differentially expresses mRNAs. Red dots represent up‐regulated mRNA and green dots represent down‐regulated mRNA

The SNP data were derived from the germ cell/somatic cell mutation site data of BC samples extracted from the second generation sequencing data using the VarScan method in the TCGA database. The search identified 517 genes that were mutated in more than 15 samples. Of the 517 genes, 20 were mutated in 50 or more samples (Figure 1). 1208 BC‐related samples about the gene expression data were obtained from the TCGA database, including 112 normal tissue samples and 1096 cancer tissue samples. And differential genetic analysis between BC and normal tissue samples was performed based on the relevant LIMMA software package. Finally, 2138 differentially expressed genes were obtained with |log FC| > 2, *P* < 0.01 as the cutoff condition (Figure 2). The dysfunctions caused by abnormal mutations and expression disorders in normal and diseased patients were explored by further analysis of these mutated genes and differentially expressed genes.

### 3.2 Functional enrichment and path analysis of mutant genes

**Figure 3:**
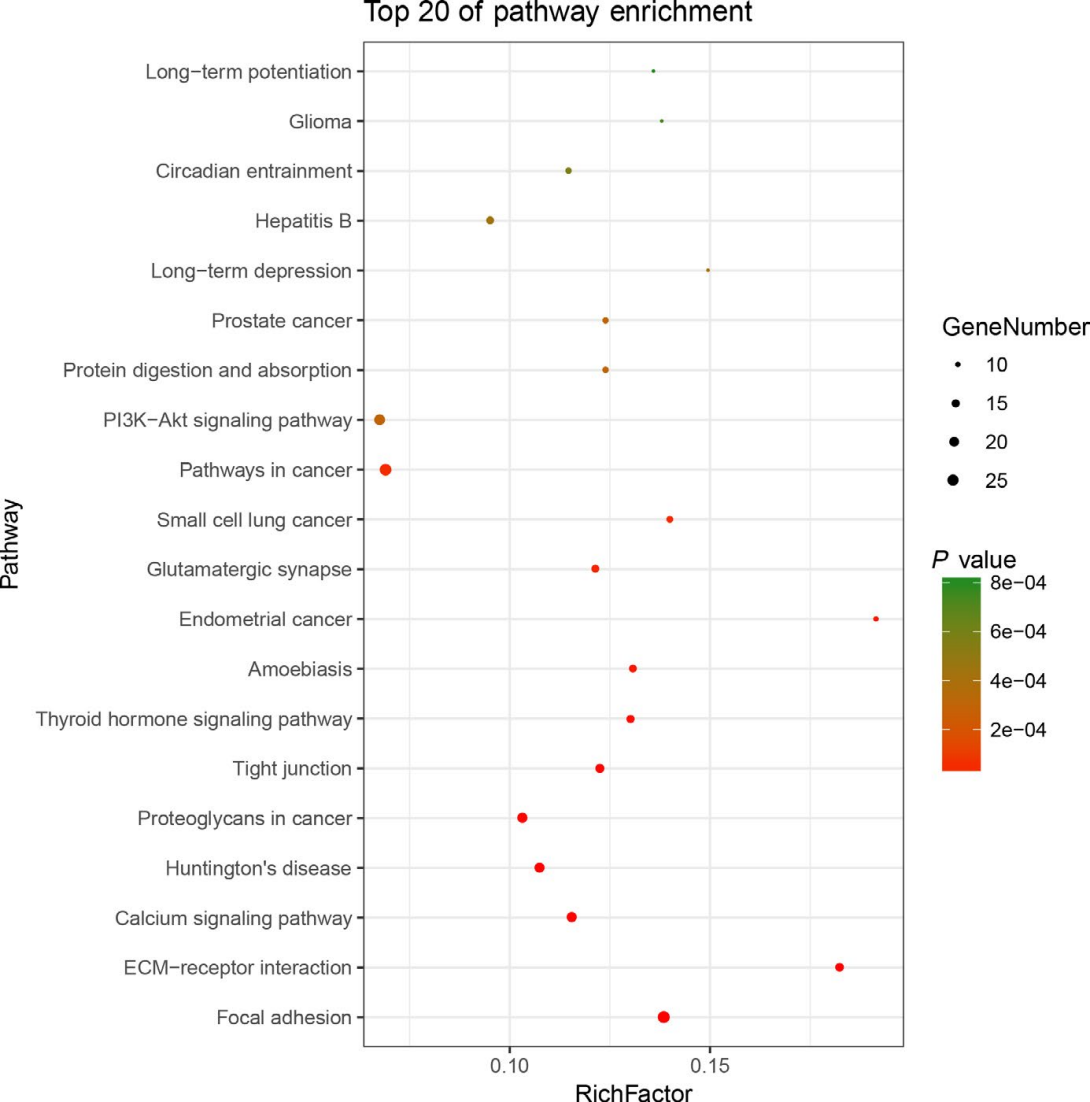
Pathways enrichment map of 517 mutant genes. The top 20 terms with the lowest P value were selected. Count: the number of enriched genes in each term

To further understand the functional role of mutant genes in BC, we used DAVID online software to perform functional enrichment analysis and pathway analysis of the 517 genes that were mutated in more than 15 samples. The abnormal mutations of genes related to BC were enriched in multiple pathways that affected MF and biological processes. Pathway analysis revealed the enrichment of SNP mutant genes in many signaling pathways in cancer, including the phosphoinositol‐3‐kinase (PI3K)‐Akt, calcium, and mitogen‐activated protein kinase (MAPK) signaling pathways, among others (Figure 3). A functional analysis revealed that in the biological process (BP) group, SNP mutant genes were mainly enriched in regulation of transcription, including positive regulation of transcription from RNA polymerase II promoter, negative regulation of transcription from RNA polymerase II promoter, and positive regulation of GTPase activity. In the MF group, these genes were mainly enriched in the binding of protein, calcium ion, and DNA. And in the CC group, these genes were particular in the cytoplasm and nucleoplasm (Table 1).

**Table 1:** Gene ontology analysis of 517 mutant genes in breast cancer

Category	Term	Count	*P* value
GOTERM_BP_DIRECT	Positive regulation of transcription from RNA polymerase II promoter	45	0.001617
	cell adhesion	38	1.04E‐08
	Negative regulation of transcription from RNA polymerase II promoter	32	0.0132158
	positive regulation of GTPase activity	28	0.0055662
	Positive regulation of transcription, DNA‐templated	27	0.0031846
	Negative regulation of transcription, DNA‐templated	25	0.0080431
	Protein phosphorylation	23	0.0104001
	Extracellular matrix organization	22	1.72E‐07
	Homophilic cell adhesion via plasma membrane adhesion molecules	20	1.08E‐07
	Axon guidance	20	1.20E‐07
GOTERM_MF_DIRECT	ATP binding	105	1.15E‐20
	Protein binding	263	0.0032137
	Calcium ion binding	61	2.12E‐15
	DNA binding	57	0.0413412
	Zinc ion binding	45	0.0120743
	Calmodulin binding	34	4.72E‐18
	ATPase activity	31	1.06E‐15
	Identical protein binding	31	0.0166445
	Actin binding	30	3.42E‐10
	Protein serine/threonine kinase activity	23	4.75E‐04
GOTERM_CC_DIRECT	Cytoplasm	189	7.46E‐06
	Plasma membrane	158	2.82E‐06
	Cytosol	108	0.0361671
	Extracellular exosome	107	3.03E‐04
	Nucleoplasm	98	0.0066943
	Membrane	93	2.12E‐05
	Microtubule	32	6.15E‐10
	Cell junction	31	1.13E‐05
	Perinuclear region of cytoplasm	27	0.0221612
	Z disc	26	1.22E‐15

Note

Top 10 terms were selected according to count and *P* value <0.05. Count: the number of enriched genes in each term.

### 3.3 Construction of mutant gene PPI network and correlation analysis of gene expression

**Figure 4:**
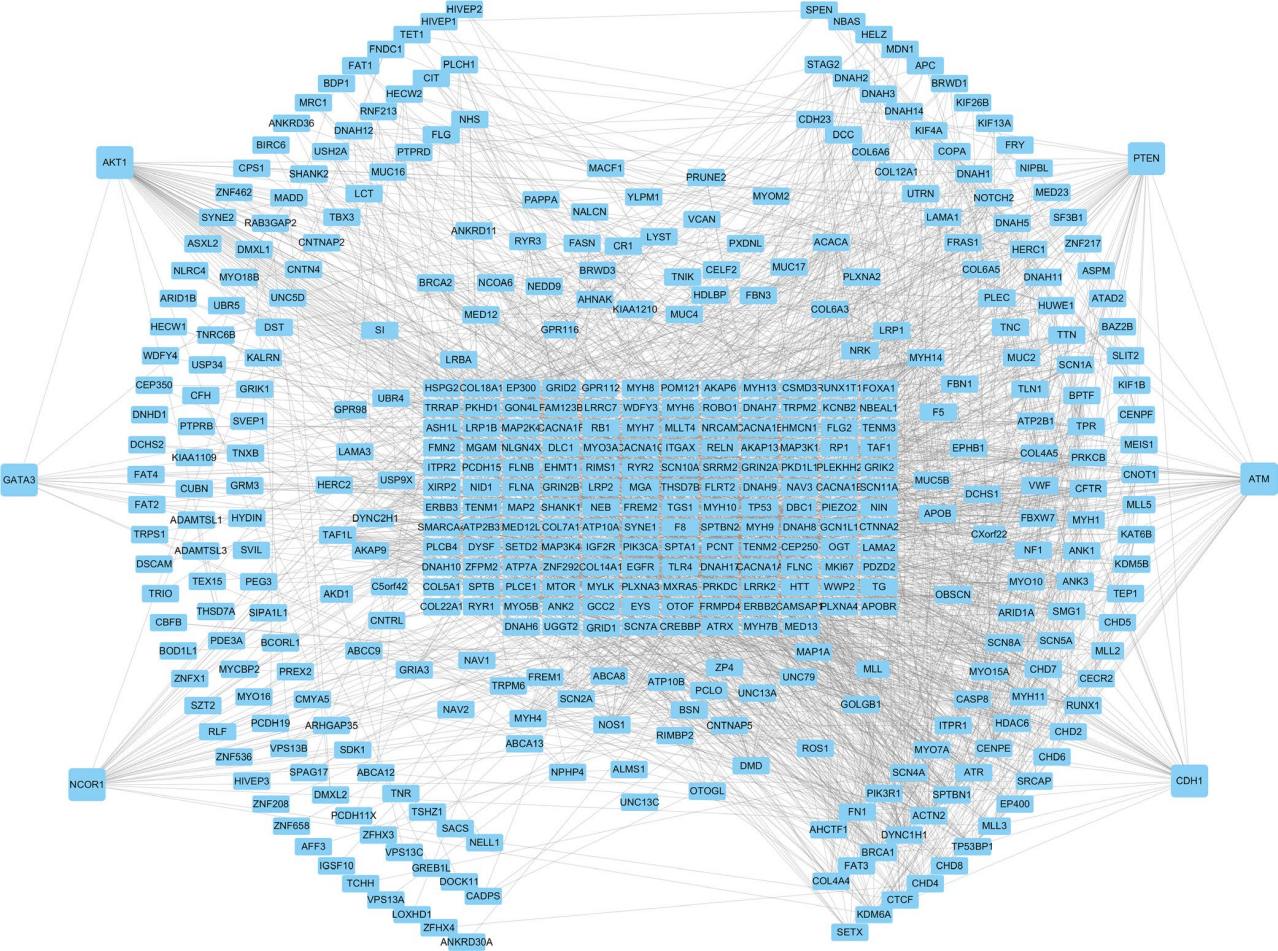
The PPI network of the 517 mutant genes in breast cancer

**Figure 5:**
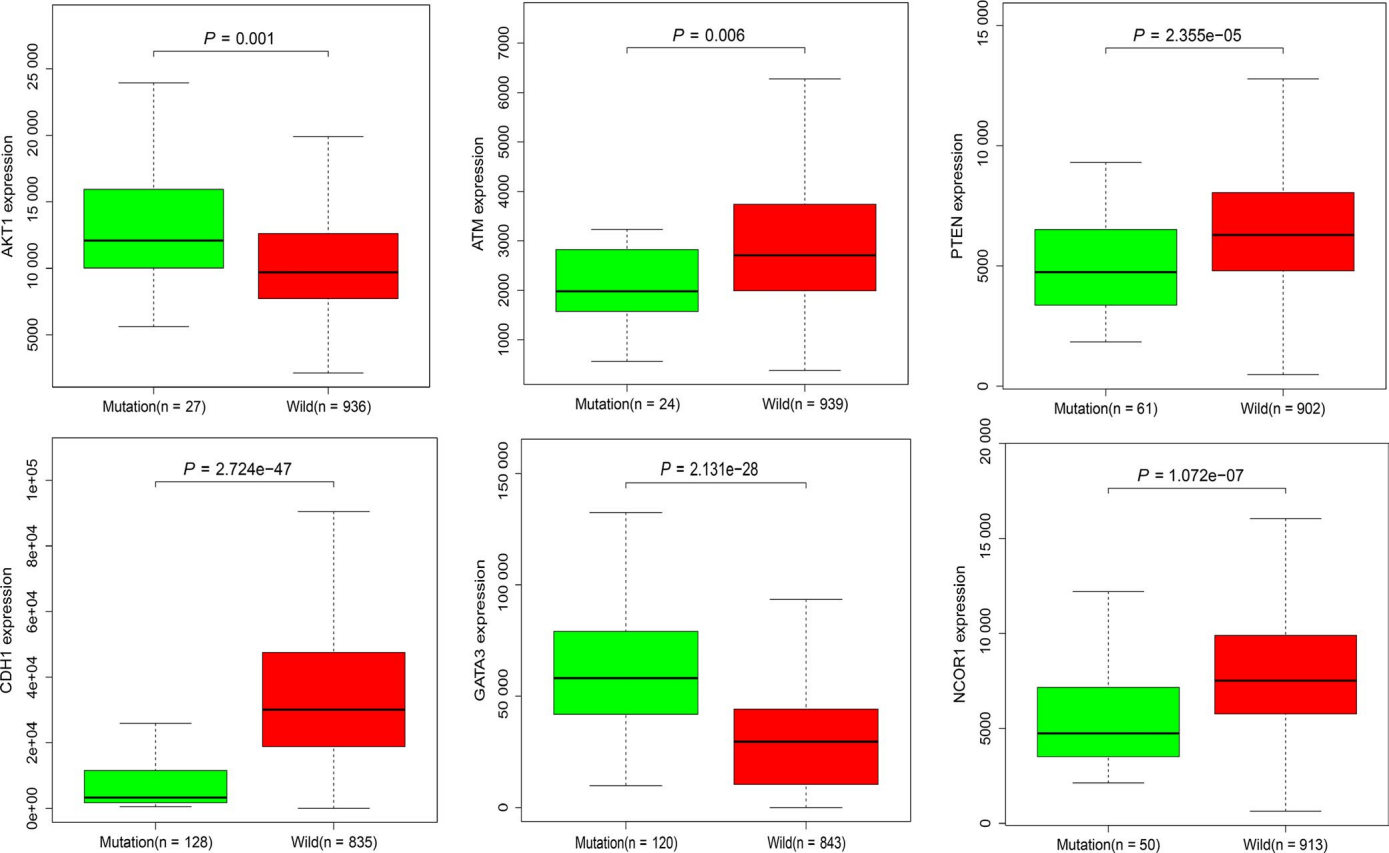
The relationship between mutation and expression about six genes

**Figure 6:**
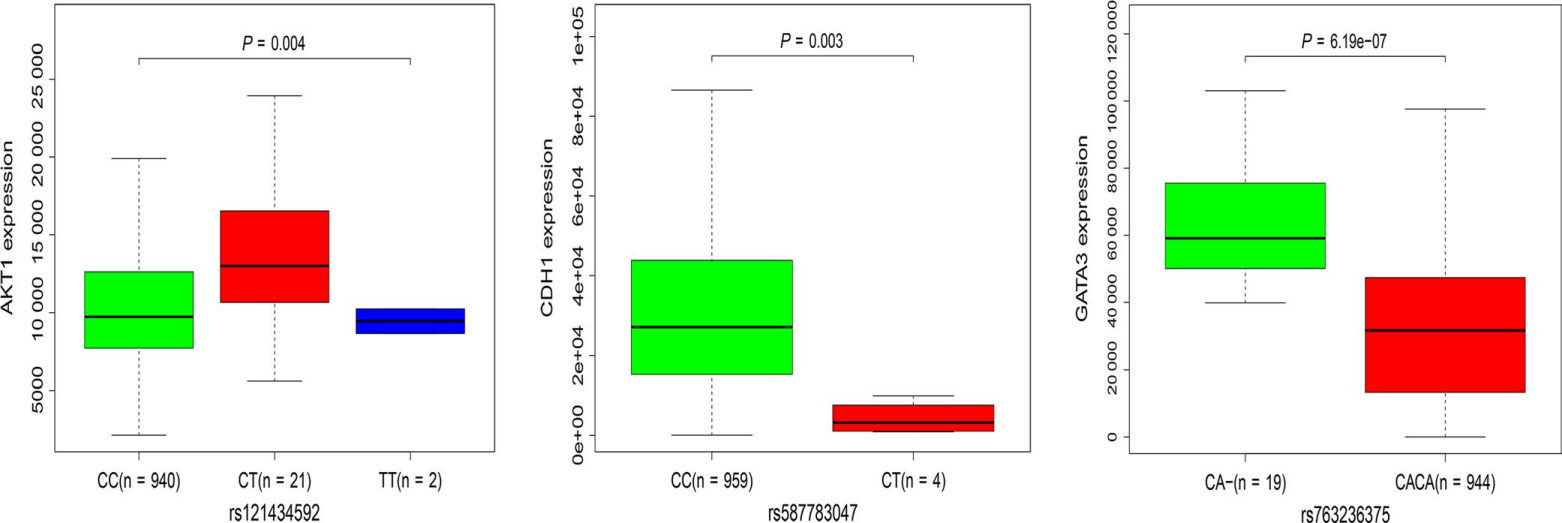
The relationship between mutation sites and corresponding gene expression of AKT1, CDH1, and GATA3

To further investigate the potential links between these mutant genes, the STRING online database was used to mine and describe the interactions between mutant genes. The complex PPI network visualized using Cytoscape software contained 447 nodes and 2553 edges (Figure 4). The correlation analysis revealed correlations between the mutation and expression of six genes (*NCOR1, GATA3, CDH1, ATM, AKT1, and PTEN*). Among them, the expression levels of *CDH1, NCOR1, ATM, *and *PTEN* in the mutant samples were decreased, while the expression of *GATA3* and *AKT1* increased (Figure 5). In addition, further site analysis revealed that mutations in AKT1 rs121434592, CDH1 rs587783047, and GATA3 rs763236375 were significantly associated with corresponding gene expression (Figure 6). Limited by the sample size, the gene NCOR1, ATM, and PTEN mutation sites samples were too small, leading to the failure to find the mutation sites that affect gene expression, and further exploration is needed later.

### 3.4 Kaplan‐Meier survival curve analysis of mutant genes and screening of prognostic biomarkers

**Figure 7:**
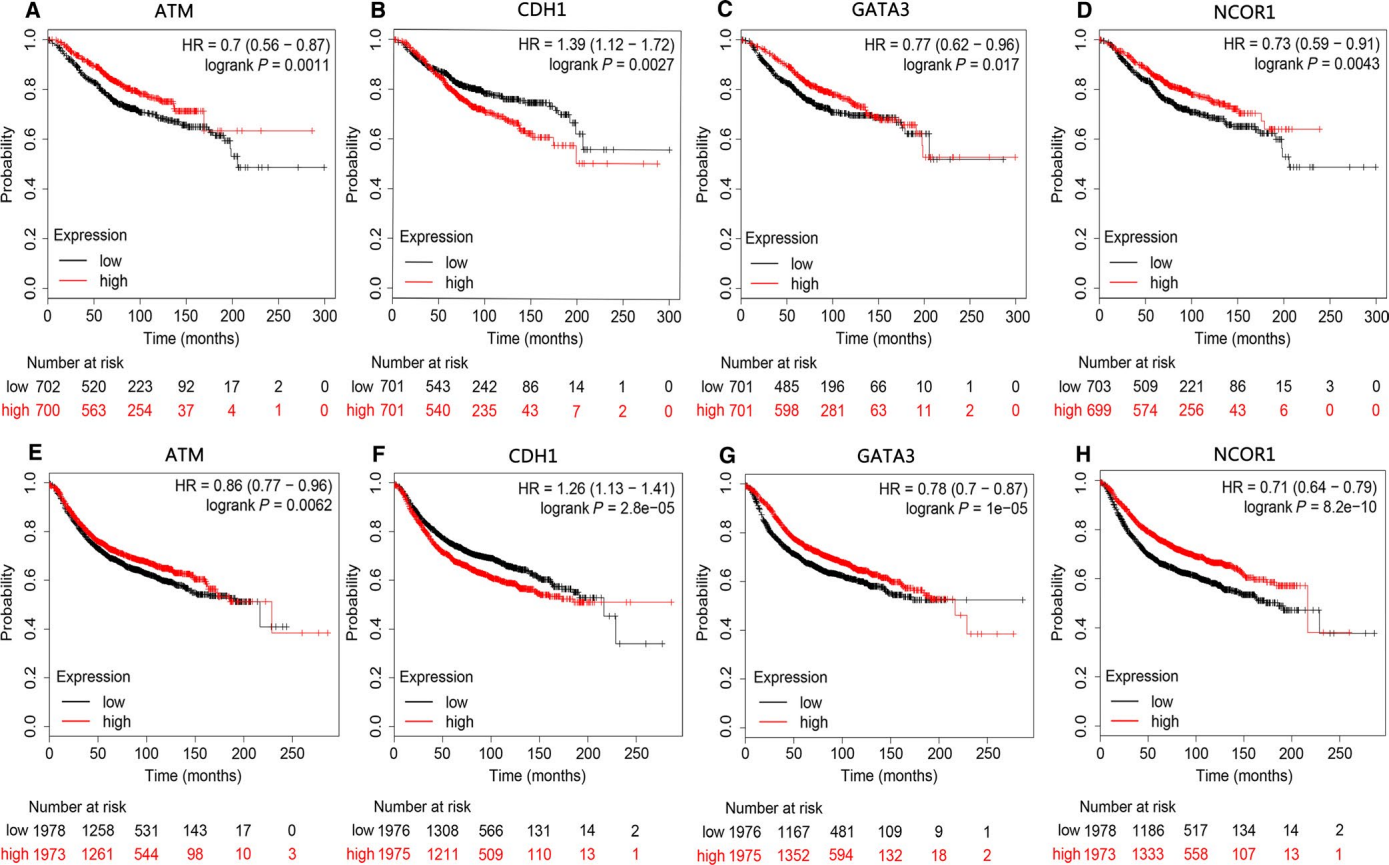
Kaplan‐Meier survival curves of the mutant genes. (A‐D) The OS curves of the mutant genes, (E‐H) The RFS curves of the mutant genes

Based on Kaplan‐Meier plots, patients were divided into high expression group and low expression group according to the median expression value. The OS and RFS curves of the six expression‐related mutant genes were plotted. Using *P* < 0.05 as the significance level, the expression of four genes (*NCOR1, GATA3, CDH1,* and* ATM*) was found to be closely related to patients' OS and RFS. The OS and RFS curves revealed that high expression of *NCOR1, ATM,* and *GATA3* was associated with higher OS and RFS, while high expression of *CHD1* was associated with poor prognosis (Figure 7).

## 4 DISCUSSION

BC is a complex disease that is the most common cause of cancer deaths in women worldwide. Detailed knowledge of the molecular pathogenesis of BC, early detection of SNP mutations, and identification of prognostic markers affecting the development of disease is needed to improve the quality of life and prognosis of patients. Polymorphisms in many genes reportedly alter the risk of cancer and are considered potential markers of carcinogenesis. Polymorphisms of matrix metalloproteinase (MMP)8 rs11225394 and MMP9 rs3787268 are closely related to BC risk in the Chinese Han population.^13^ The XPG (rs1047768 T > C) mutation may play an important role in the reduction of progression‐free survival and can be used as a predictor of poor prognosis in BC.^14^ Therefore, bioinformatics analysis of the prognosis of mutated genes and screening for specific SNP mutated genes can provide clinicians with new tools for treating patients and predicting prognosis.

In the present study, our aim was to screen and identify prognostic biomarkers associated with SNP‐mediated expression through a series of bioinformatics analyses of BC‐related data in TCGA database. To further study the related molecular mechanisms involved in the direct involvement of these mutant genes, functional enrichment and pathway analysis were performed. The genes were enriched in the cytoplasm and nucleoplasm, and were mainly involved in certain transcriptional regulation, such as negative or positive regulation of transcription from RNA polymerase II promoter, positive regulation of transcription from DNA templates, and various binding pathways, such as protein and DNA binding. Pathway analysis indicated that BC mutated genes were mainly involved in the PI3K‐Akt and, calcium signaling pathways, and many other pathways related to cancer development. The functional enrichment and pathway analyses demonstrate the molecular mechanisms of SNP mutations in disease progression, and the functional level interaction of these genes.

Mutations in six genes (*NCOR1, GATA3, CDH1, ATM, AKT1, *and *PTEN*) were significantly correlated with the corresponding expression levels, and were enriched and involved in multiple cancer‐related pathways. GATA binding protein 3 (GATA3) is a transcription factor crucial for mammary gland morphology and cell differentiation and acts as a tumor suppressor.^15^ Studies by Dydensborg et al have shown that overexpression of *GATA3* could inhibit tumor growth and lung metastasis.^16^ Presently, *GATA3* SNP mutations were identified in BC samples, and were positively correlated with expression levels, that is, the expression level of *GATA3* was also increased in mutant samples. In addition, studies by Atlas et al have confirmed that the *GATA3* gene is identified with mutations in >10% of all BC samples,^17^ further indicating that our results are feasible and accuracy. In addition, further analysis indicated that the SNP mutation in CACA at the GATA3 rs763236375 site was the important reason for affecting gene expression. The OS and RFS analysis indicates that high expression of this gene is beneficial to the prognosis of BC patients.

PI3K/AKT is an important signal transduction pathway in cells, which is significantly associated with malignant tumor metastasis.^18^ AKT is a direct target protein downstream of PI3K; increasing evidence supports the view that activation of AKT protein has an important biological role in cancer development.^19^ *AKT1* is one of the subtypes of AKT. Activated *AKT1* phosphorylates a large number of downstream substrates and is involved in the regulation of cell growth, metabolism, proliferation, apoptosis, and other processes. Castaneda et al found that *AKT1* is closely related to the early development of BC^22^ and can be used as a key indicator for early diagnosis of BC. Pathway analysis revealed that *AKT1* is enriched in the PI3K‐AKT signaling pathway and in several other pathways closely related to cancer, such as proteoglycans in cancer and the MAPK signaling pathway, indicating that the gene encoding *AKT1* has important biological functions in the development of cancer. Moreover, our study also found that the important reason for the correlation between AKT1 gene SNP mutation and expression is the mutation of CC in AKT1 rs121434592 site. Furthermore, as a guardian of genomic integrity, the tumor suppressor gene *PTEN* plays an important role in maintaining chromosomal stability.^23^ *PTEN* is absent in most BC patients, especially triple‐negative BC. The loss of *PTEN* and phosphorylation activate AKT, and the activation regulates the PI3K/AKT pathway, which affects BC progression and patient prognosis.^26^

Nuclear receptor corepressor 1 (*NCOR1*) is a transcriptional co‐regulator that binds chromatin‐modifying enzymes to gene‐specific transcription factors and interacts with members of the BTB‐ZF transcription factor family to play important roles in T cell development and function. Low expression of *NCOR1* is associated with acquired tamoxifen resistance in a mouse model of BC.^29^ Recent data have also shown that decreased *NCOR1* expression is significantly associated with shorter RFS in BC patients, suggesting a poor prognosis^30^ that may be related to immune system involvement and enhanced drug resistance. In our study, the expression of the *NCOR1* gene was significantly reduced in the mutated samples, and the results of the correlation analysis showed that the SNP mutation of *NCOR1* was negatively correlated with the expression level. Moreover, the OS and RFS analysis data supported the poor prognosis associated with low expression of *NCOR1*, which is consistent with previous studies and further confirms the validity of the present findings.

E‐cadherin (*CDH1*) and Ataxia telangiectasia mutated (*ATM*) are tumor suppressor genes, which are enriched in multiple signaling pathways, including cancer pathways, miRNAs in cancer, apoptosis, and the p53 signaling pathway. *CDH1* is frequently mutated in diffuse gastric cancer and lobular BC. Diffuse gastric cancer patients with *CDH1* mutation have shorter survival time than those without the *CDH1* mutation.^33^ In addition, mutations in *ATM* are closely associated with BC, ovarian cancer, and other cancers.^34^ *ATM* expression is down‐regulated in BC and suggests poor prognosis.^35^ Hypermethylation of the *ATM* gene promoter might affect the DNA repair mechanism by causing the dysregulation of the ATM/p53 signaling pathway, thereby affecting tumor progression in BC.^36^ In our study, the results of the correlation analysis showed that the SNP mutations of the genes encoding *CDH1* and *ATM* were negatively correlated with the expression levels, with SNPs resulting in reduced expression. Among them, the main reason for the correlation between CDH1 SNP mutation and expression is the mutation of CC in CDH1 rs587783047 site. In addition, the OS and RFS analysis revealed that decreased expression of *ATM* detrimentally affects the prognosis of patients. Conversely, the increased expression of *CDH1* detrimentally affects the prognosis of patients, which required further clinical investigation.

## 5 CONCLUSION

Bioinformatics analysis revealed that SNPs in six genes (*NCOR1, GATA3, CDH1, ATM, AKT1, *and *PTEN*) were significantly associated with the corresponding expression levels and were involved in multiple pathways involved in cancer development. In addition, further analysis indicated that the SNP mutation at the AKT1 rs121434592, CDH1 rs587783047, and GATA3 rs763236375 sites were the important reasons for affecting gene expression. In addition, OS and RFS analysis found that the expression of *NCOR1, GATA3, CDH1, *and *ATM* were closely related to the survival of BC patients. Therefore, detecting gene mutations and exploring their corresponding expression can be used to predict the prognosis of patients. The findings will require validation in large‐scale clinical studies to determine their accuracy and sensitivity in tumorigenesis and predicting patient outcomes. However, the focus of this study is to provide new ideas for clinical diagnosis and evaluation of prognosis through bioinformatics analysis. Our results provide an important bioinformatics basis and relevant theoretical basis for guiding follow‐up studies on BC.

## CONFLICT OF INTEREST

The authors declare that they have no competing interests.
